# Metabolomics-guided identification of bioactive phytometabolites from South African plants targeting neuroblastoma

**DOI:** 10.3389/ebm.2026.10867

**Published:** 2026-03-05

**Authors:** Mmei Cheryl Motshudi, Clarissa Marcelle Naidoo, Chikwelu Lawrence Obi, Benson Chucks Iweriebor, Earl Prinsloo, Muhammad Sulaiman Zubair, Nqobile Monate Mkolo

**Affiliations:** 1 Department of Biology, School of Science and Technology, Sefako Makgatho Health Science University, Pretoria, South Africa; 2 Department of Biotechnology, Rhodes University, Makhanda, South Africa; 3 Department of Pharmacy, University of Tadulako, Palu, Indonesia

**Keywords:** *Acorus calamus*, ADME, *Lippia javanica*, metabolomics, mitochondrial membrane potential

## Abstract

Neuroblastoma constitutes a solid tumor in pediatric populations, characterized by a dismal prognosis and a scarcity of effective therapeutic interventions. Medicinal flora from South Africa represents valuable sources of bioactive phytometabolites with potential relevance to neuroblastoma. This study employed an integrated workflow merging untargeted UPLC-MS/MS metabolomics, mitochondrial functional assays, and *in silico* absorption, distribution, metabolism, and excretion (ADME) prediction to systematically identify bioactive metabolites from *Acorus calamus* and *Lippia javanica* with activity against SH-SY5Y neuroblastoma cells. Cytotoxic effects were quantified utilizing the CCK-8 assay, while mitochondrial membrane potential (ΔΨm) was conducted through JC-1 flow cytometry. Untargeted UPLC-MS/MS profiling yielded metabolomic fingerprints, through PCA, PLS-DA, and OPLS-DA. ADME and drug-likeness were predicted using SWISSADME. Both plant extracts exhibited dose-dependent inhibition of SH-SY5Y cell viability, with IC_50_ values determined at 0.2886 μg/μL for *A. calamus* and 0.3066 μg/μL for *L. javanica*. The ΔΨm assessment indicated enhanced mitochondrial polarization (68.2% and 65.4% compared to 58.8% in untreated controls), implying modulation of mitochondrial functional status. Metabolomic profiling unveiled distinct phytochemical signatures, including flavonoids, phenolics, jasmonates, and alkaloids, exhibiting significant species-level differentiation (*F* = 936.71, *R* ^*2*^ = 0.989, *p* = 0.005). Notable metabolites such as isopropyl β-glucoside, 6β-hydroxymethandienone, and 7-epi-12-hydroxyjasmonic acid demonstrated favorable ADME characteristics and permeability across the blood-brain barrier. This investigation elucidates that *A. calamus* and *L. javanica* possess potential efficacy against neuroblastoma, underscoring the translational potential of African medicinal flora in pediatric oncology and necessitating further preclinical exploration.

## Impact statement

This manuscript serves to investigate the medicinal flora for natural bioactive compounds as probable sources of therapeutic anticancer agents targeting SH-SY5Y neuroblastoma cells. Utilizing a multifaceted methodology that incorporates cytotoxicity assays, untargeted metabolomics, and in silico Absorption, Distribution, Metabolism, and Excretion modeling, we have identified phytometabolites exhibiting promising anticancer efficacy and have also predicted their permeability across the blood–brain barrier. The findings will offer different perspectives and insights into the metabolomic profiling and therapeutic potential of the medicinal plants, thereby enhancing the pharmacological comprehension of phytochemical agents in pediatric oncology.

This manuscript also highlights the current strategic plans for neuroblastoma treatment and approaches that could be applied in the future to improve the quality of life of patients with neuroblastoma, as well as to provide early diagnostic measures to reduce mortality rates of this fatal cancer. This information could be of great use for both clinical and scientific research.

## Introduction

Neuroblastoma (NB) is a primary cancer detected in infants; it is a predominant solid tumor commonly found in the extracranial area in children [1]. Neuroblastoma accounts for approximately 10–15% of childhood malignancies, and the primary tumour generally stems from the sympathetic chain, usually in the abdomen or in the adrenal gland [2]. Patients with neuroblastoma tend to exhibit a wide range of biological, clinical, accompanied by prognostic heterogeneity, and a fundamental prognostic trait pertaining to neuroblastoma patients is the location of the primary tumour, which can originate in the adrenal gland, abdominal/retroperitoneal area, neck, thorax, pelvis, or, less frequently, in other sites [3, 4]. Due to the heterogeneous biological nature of neuroblastoma, its prognosis, along with clinical course, differs between spontaneous regression to high-risk cases, with several neuroblastoma tumours exhibit poorly responsive to intensive multimodal therapy [5, 6]. Presently treatment strategies involve aggressive amalgamation of radiotherapy, induction chemotherapy, elevated-dose chemotherapy with autologous stem-cell rescue, surgical removal of tumors, and applications of post-consolidation methods such as immunotherapy, as well as differentiation therapy [7]. The 5-year overall survival rate for high-risk neuroblastoma individuals continues to hover around 50–60% despite these integrated multimodal approaches, and late effects are usually imminent and frequently experienced by long-term survivors [8]. Regardless of advancement in multimodal therapy, outcomes for high-risk neuroblastoma persist to be suboptimal, highlighting the necessity for alternative and complementary therapeutic strategies.

The SH-SY5Y human neuroblastoma cell line comprises an N-type catecholaminergic subclone of neuroblastoma commonly used in research areas pertaining to neuroblastoma and neurobiology [9]. SH-SY5Y cell lines convey crucial elements of human catecholaminergic systems, which encompass components such as dopamine-β-hydroxylase and tyrosine hydroxylase [10]. Induced SHSY5Y cells can be differentiated into phenotypes that resemble neurons, therefore rendering them an adaptable *in vitro* model [11]. However, limitations such as inadequate maturation levels of neurons and restricted functionality of network systems, irrespective of the differentiation, emphasize the necessity of cautious interpretation of pharmacological responses [12]. Changes in mitochondrial membrane potential (ΔΨm) and cellular redox balance can play context-dependent roles in cancer biology, acting as regulators of cell survival and cell death conditional on cellular state and therapeutic pressure [13]. Although moderate stabilization of mitochondrial function and redox homeostasis might permit tumour cell adaptation, excessive or dysregulated perturbation of these pathways can encourage cytotoxic or cytostatic outcomes via mitochondrial dysfunction [14, 15]. Accordingly, modulation of ΔΨm and redox-related processes has been explored as a functional vulnerability in cancer cells, also neuroblastoma, instead as a unidirectional pro-survival mechanism [16, 17].

Approximately 65% of the world’s population, along with an estimated 80% of the population in Africa as well as Asia, depends on traditional herbal remedies to cure and furthermore prevent infectious ailments and chronic diseases [18, 19]. Ethnobotanical knowledge from southern Africa has showcased many species of aromatic origin known for their anticancer, anti-inflammatory, and anti-infective properties [20]. South African based medicinal plants are rich sources of structurally diverse phytometabolites with documented biological activities, and systematic investigation of these resources might permit the discovery of candidate anti-cancer chemotypes or complementary approaches to present therapies [21–25]. *Lippia javanica* (Burm.f.) is a multi-branched woody shrub, that is commonly known as fever tea [9, 26]. *L. javanica* (Verbenaceae family) is an aromatic medicinal plant indigenous to eastern and southern Africa that has been broadly explored for its phytochemical composition and biological activities [26–30]. Previous studies have recognized phenolic, terpenoids, and flavonoids constituents, with apigenin- and luteolin-associated derivatives, which *in vitro* exhibit antioxidant and cytotoxic activities, supporting its importance for further assessment within a metabolomics-directed anticancer framework [31–36]. *Acorus calamus* Linn., (Aceraceae family), is classified as a perennial, semi-aquatic herb, that is commonly known as sweet flag {25]. *A. calamus* is an aromatic medicinal plant that has been extensively studied for its phytochemical diversity and biological activities, and it is frequently utilized in traditional Chinese and Indian medicine [37–41]. Notably, this plant comprises phenylpropanoid constituents for instance α- and β-asarone, for which extracts and isolated compounds have established anticancer activity in preclinical cancer models, also permitting its inclusion in metabolomics-directed anticancer investigations [42, 43]. Untargeted LC-MS metabolomics is commonly utilized to map systematic methods for enhancing quality control, distinguishing chemotypes, associating chemical properties with bioactivity of samples, and prioritizing key metabolites for further pharmacology analysis, alongside *in silico* ADME assessment through tools like SwissADME and the BOILED-Egg model [44–47].

Building on the above insight, while the phytochemistry and bioactivity of *L. javanica* and *A. calamus* have been previously reported in various biological contexts, their assessment within an integrated framework relating metabolomic composition to mitochondrial functional outcomes and pharmacokinetic possibility remains limited. In this study, we integrated untargeted UPLC-MS/MS metabolomics, mitochondrial membrane potential evaluation in SH-SY5Y neuroblastoma cells, and *in silico* ADME analysis to select metabolites with possible neuroblastoma importance.

## Materials and methods

### Plant collection and identification

Specimens of *L. javanica* and *A. calamus* were collected from Hartbeespoort in the North-West Province of South Africa (25.7236° S, 27.9653° E) in February 2023. Organ-matched sampling was applied to follow metabolomics reporting recommendations that highlight control of tissue-specific variability in comparative chemical analyses [48]. Species identification of both plants was authenticated by taxonomists at the National Herbarium, where voucher specimens were deposited under accession numbers NR 904 (*L. javanica*) and NR 905 (*A. calamus*). Plant material collection was completed in accordance with national regulatory requirements, under permit CF6-0234 released by the Department of Agriculture and Rural Development-Nature Conservation, South Africa (Permit Holder: Prof. Nqobile Monate Mkolo).

### Anti-cancer assay analysis

#### Sample preparation

The *L. javanica* and *A. calamus* samples were pulverized into a powder using a mortar and pestle under liquid nitrogen. A precise weight of 0.5 g from each powdered sample was combined into a single 50 mL centrifuge tube. A total of 10 mL of buffer was added to the tube followed by extraction at a 4 °C. The extractant was filtered through a 0.45 μm filter, and the clearer supernatants were transferred into a new centrifuge tube. Crude extracts and intermediate preparations were stored at −20 °C in amber, light-protected containers to reduce degradation. Extracts were permitted to equilibrate to room temperature and were used within 1 week of preparation to allow stability and duplicability.

#### SH-SY5Y cell preparation

Human neuroblastoma SH-SY5Y cells (Cellonex ™, Johannesburg South Africa) were cultured in Dulbecco’s Modified Eagle’s Medium (DMEM) supplemented with 10% (v/v) heat-inactivated fetal bovine serum, 1% penicillin-streptomycin obtained from Gibco (New York, NY, United States), and maintained at 37 °C in a humidified incubator with 5% CO_2_ [49]. Cells were washed by utilizing a 1 mL pipette to aspirate and discard the old medium. Gradually, 2 mL of Phosphate Buffered Saline (PBS) from Sigma-Aldrich (St. Louis, MO, United States), was poured along the edge of the T25 flask, and this flask was gently swirled to wash away any remaining medium. Cell digestion was conducted by gradually adding 1 mL of 0.25% trypsin digest solution (Sigma-Aldrich, St. Louis, MO, United States) into the T25 flask, ensuring it covered all the cells. The flask was then placed in a 37 °C CO_2_ incubator for 2 min. Cells were examined, using an inverted microscope, for separation and formation of a spherical shape. To stop digestion, 4 mL of cell growth medium was added to the cells. Passage was done at a ratio of 1:4, where the cells were carefully pipetted for dispensation, and a single-cell suspension was confirmed under the microscope.

#### CCK-8 assay

The effect of chosen plant tissue (final concentration of 0.01–10 μg/mL) on SH-SY5Y cell viability was assessed using the CCK-8 assay according to the protocol [50]. SH-SY5Y cells were collected from an approximately 80% confluent T-25 flask using the standard trypsinization method. Afterward, 2 × 10^4^ cells were seeded in a 96-well plate and incubated for 24 h at 37 °C. Then, the media was removed, rinsed with PBS, and the cells were treated with 100 μL of clear supernatants from the sample, while the cell medium served as the negative control. Following 24 h, 10 μL of CCK-8 was added to every well and allowed to incubate for 2 h. A microplate reader (Infinite M200, Tecan, Switzerland) scanned the 96-well plate at a wavelength of 450 nm. All CCK-8 experiments were performed utilizing three independent biological replicates, with each condition examined in technical triplicate. The subsequent formula was applied to determine the percentage of cell viability:

$$\text{Survival rate }\left(\%\right)=\left(\text{Asample}-\text{Ab}/\text{Ac}-\text{Ab}\right)\times 100$$

Wherein Asample, Ab, and Ac are the absorbance/Optical density of cells exposed to test reagents, absorbance/Optical density of blank, and negative control, respectively.

#### Mitochondrial membrane potential detection

The mitochondrial membrane potential (ΔΨm) of SHSY-5Y cells against *L. javanica* and *A. calamus* was determined by adopting a procedure of Sakamuru and co-workers [51]. SH-SY5Y cells were seeded in a 96-well plate (2 × 10^4^ cells per well) according to experimental groups, which included the blank control group (non-treated SH-SYSY cell line), negative control of non-treated SH-SYSY cell line for gating, and the treated SH-SYSY cell line group (*L. javanica* and *A. calamus* at 1 µg/uL). After 24 h of treatment, the cells were collected using trypsin for adherent cells to avoid over-digestion and to prevent membrane potential damage. A JC-1 working solution was prepared using a diluted JC-1 stock solution with pre-warmed (37 °C) medium, which was added to the final concentration. The JC-1 working solution was then added to the cells and incubated at 37 °C in the dark for 15 min. JC-1 solution was removed, and cells were washed 3 times with pre-cooled PBS to remove unbound dye. The cells were collected using trypsin, then centrifuged and resuspended into PBS. The set flow cytometry channels were green fluorescence, which was the FL1 channel (Ex 488 nm, Em 530/30 nm), and red fluorescence, which was the FL2 channel (Ex 488 nm, Em 585/42 nm). The red fluorescence (normal mitochondria) and green fluorescence (decreased membrane potential) were then merged for analysis. Mitochondrial membrane potential (ΔΨm) assessments were conducted utilizing three independent biological replicates, with technical triplicates for each experimental condition.

### Metabolomic characterization

#### Chemicals and instrumentation

Multiple chemicals and reagents, such as acetonitrile (Merck, Rahway, NJ, United States), methanol (Merck, Rahway, NJ, United States), DL-o-Chlorophenylalanine (Merck, Rahway, NJ, United States), and formic acid (Merck, Rahway, NJ, United States) were utilized. Additionally, instrumentation, namely ACQUITY UPLC HSS T3 (100 × 2.1 mm × 1.8 μm), Ultimate 3000LC combined with Q Exactive MS (Thermo, Waltham, MA, United States), and Temp functional Centrifugation (Eppendorf, Enfield, CT, United States) were utilized.

#### Sample preparation

The initial stages of the sample preparation comprised a freeze-drying process (lyophilization) of *A. calamus* and *L. javanica* leaf samples. The respective samples were subsequently dried and ground into powder; the resultant powder for each sample was transferred into a 5 mL homogenizing tube. A MM 400 mixer with four 5 mm diameter metal balls was utilized to mix the samples at 30 Hz. The subsequent product for each sample was added with 80% methanol at a volume of 800 µL. The resultant solution was vortexed for 30 s and sonicated thereafter for 30 min at 4 °C. Samples were stored at a temperature of −20 °C for an hour. Each sample was centrifuged thereafter at 12,000 rpm, 4 °C for 5 min. A total of 200 µL of each supernatant from the respective samples, along with 5 µL of DL-o-Chlorophenylalanine (140 μg/mL), were added to vials. The vials were then used for liquid chromatography–mass spectroscopy (LC-MS) analysis.

#### Untargeted plant metabolomics analysis using UPLC-MS/MS

Separation of compounds was carried out using the Ultimate 3000LC (Thermo, Waltham, MA, United States) combined with QExactive MS (Thermo) and additionally, screened with ESI-MS [27]. The first solvent of the mobile phase comprised 0.05% formic acid in water and the second solvent was acetonitrile with a subsequent gradient elution (0–1 min, 95% A; 1–12 min, 95%–5% A; 12–13.5 min, 5% A; 13.5–13.6 min, 5–95% A; 13.6–16 min, 95% A). The mobile phase had a flow rate of 0.3 mL·min^−1^. The column temperature was kept at 40 °C, while the sample manager temperature was adjusted to 4 °C. The mass spectrometry settings for electrospray ionization ESI+ mode are detailed as follows: heater temperature 300 °C; sheath gas flow rate, 45 arb; auxiliary gas flow rate, 15 arb; sweep gas flow rate, 1 arb; spray voltage, 3.0 kV; capillary temperature, 350 °C; S-Lens RF level, 30%.

#### Quality control samples

Extracts of the respective plants were mixed as quality control samples (QC) to evaluate the methodology and stability of the LC-MS system. Raw data for the UPLC–MS/MS were obtained from Compound Discover (3.0, Thermo) based on the m/z value and the retention time of the ion signals. To ensure consistency and efficacy of the system, multiple QC samples were formulated through a combination of identical quantities of each isolated sample. The instrument’s performance and repeatability were evaluated through pooled QC samples that were injected before the sample analysis until equilibrium was reached. The QC samples were subsequently run in positive mode. The ion characteristics of the QC samples were utilized to determine the Relative Standard Deviation (RSD). Supplementary Figure S1 of the Supplementary Material displays the percentage of RSD distribution, with a significant portion of the RSD being below 30%. This indicates that the analytical method is reliable and appropriate for application to future sample analyses.

#### Identification of metabolites

Metabolite identification was performed at a putative level (MSI level 2) utilizing accurate mass measurements and MS/MS fragmentation data. Candidate metabolites were annotated by matching experimental spectra against the Human Metabolome Database^1^, ChemSpider^2^ and MassBank^3^, all accessed 19 August 2024. Then manual evaluation of retention time consistency, isotope patterns, and MS/MS fragmentation spectra was done. Cross-validation across databases was achieved to enhance annotation confidence. Features detected in the ESI^+^ mode were cross-checked for chromatographic and spectral consistency and combined where appropriate. Redundant ions and adducts were resolved utilizing mass accuracy criteria (<5 ppm), retention-time coherence, signal intensity, and % RSD across technical replicates to reduce duplication or erroneous assignments.

### Pharmacokinetic and drug-likeness activity of differential metabolites

The properties of the ADME of significant metabolites were extrapolated through *in silico* methods, and their potential, along with their suitability as drug candidates, was evaluated. The SWISSADME platform^4^ (accessed on 12 July 2025) was utilized for primary screening, which facilitated the high ranking of compounds that exhibited drug-like profiles and advantageous pharmacokinetic properties. Subsequently, further assessments were carried out with the assistance of recognized cheminformatics inclined tools for a more thorough evaluation of the pharmaceutical relevance of the metabolites. The parameters examined comprised physicochemical descriptors, which included molecular weight, number of heavy aromatic atoms, topological polar surface area (TPSA), molecular refractivity, number of hydrogen bonds, lipophilicity factors (logP), water solubility (logS), along other parameters were examined. Pharmacokinetic traits such as blood–brain barrier (BBB) permeation, gastrointestinal (GI) absorption, substrate specificity for P-glycoprotein (P-gp), inhibition potential essential key cytochrome P450 (CYP) isoenzymes (CYP1A2, CYP2C19, CYP2C9, CYP2D6, and CYP3A4), as well as skin permeation (logKp) were evaluated, and these aspects conjointly affect the metabolism of the potential and the likelihood of drug-to-drug interactions. Ultimately, the final analysis for drug-likeness was conducted using Lipinski, Ghose, Veber, Egan, Muegge, and bioavailability scores. These assessments provide a comprehensive view of the compounds’ appropriateness for further progression.

### Statistical analysis

In terms of anti-cancer analysis, GraphPad Prism version 8.2.0 (GraphPad Software, Inc., San Diego, CA, United States) was utilized to plot the dose-response curve and determine the IC_50_ of plant treatments.

While for metabolomics analysis, the raw data was collected and aligned utilizing Compound Discover (3.0, Thermo) according to the m/z values and the retention time of the ion signals. Ions from both ESI+ were combined and subsequently transferred into the SIMCA-P software (version 14.1) and Metaboanalyst version 6.0^5^ (accessed on 12 August 2025) for multivariate analysis. Unsupervised principal component analysis (PCA) was achieved to evaluate intrinsic clusters and the structure of variance. Supervised partial least squares-discriminant analysis (PLS-DA) and orthogonal PLS-DA (OPLS-DA) were also achieved for maximizing class segregation and assessing statistically significant metabolites. Cross-validation of these models was completed, and the variance percentages clarified through orthogonal and predictive components were verified.

Statistically significant metabolites were verified and filtered by integrating the outcomes of the Variable Importance in Projection (VIP) score greater than 1.5, a *p*-value less than 0.05, FDR-adjusted *p* < 0.05 and a fold change (FC) greater than 2.0. The *R* ^*2*^ and *Q* ^*2*^ values can explain the quality of the fitted model. *R* ^*2*^ shows the variance accounted for in the model and reflects the goodness of the fit. *Q* ^*2*^ shows the variability in the data, reflecting the model’s predictability.

## Results

### Anti-cancer activities

**FIGURE 1:**
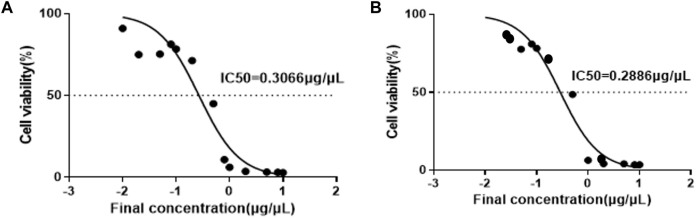
Dose-response curves for **(A)** *A. calamus* and **(B)** *L. javanica* extracts on SH-SY5Y neuroblastoma cells utilizing the CCK-8 assay.

The anti-cancer activities of *A. calamus* and *L. javanica* extracts on SH-SY5Y neuroblastoma cells were assessed utilizing the CCK-8 assay. These plant extracts displayed a clear dose-dependent decrease in cell viability, as demonstrated by the sigmoidal dose-response curves (Figure 1). The estimated half maximal inhibitory concentration (IC_50_) values were 0.2886 μg/μL for *A. calamus* and 0.3066 μg/μL for *L. javanica*, with 0.2976 μg/μL as an overall IC_50_ mean. However, *A. calamus* displayed slightly higher effectiveness in comparison to *L. javanica.*

### Mitochondrial membrane potential analysis

**FIGURE 2:**
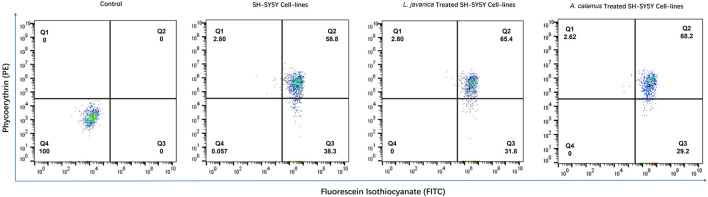
Mitochondrial membrane potential (ΔΨm) analysis of SH-SY5Y neuroblastoma cells using the JC-1 fluorescent probe and flow cytometry.

Mitochondrial function in SH-SY5Y neuroblastoma cells was evaluated using flow cytometry and the JC-1 fluorescent probe to quantify variations in membrane potential (ΔΨm). As illustrated in Figure 2, in terms of untreated SH-SY5Y control cells, 58.8% of the population was within the Q2 gate, signifying cells with high ΔΨm (red fluorescence), although 38.3% of the population was situated in the Q3 gate, conforming to mitochondrial depolarization (green fluorescence). Following plant extract treatment, the proportion of the cells with high ΔΨm increased to 68.2% for *A. calamus* and 65.4% for *L. javanica*, implying increased mitochondrial polarization comparative to untreated controls.

### UPLC-MS/MS base peak intensity chromatograms

**FIGURE 3:**
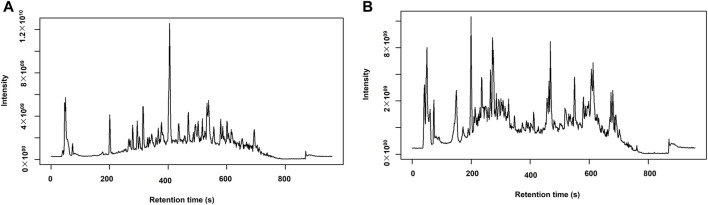
Base peak intensity (BPI) chromatograms from **(A)** *A. calamus* and **(B)** *L. javanica* in ESI+ mode.

The UPLC-MS/MS analysis of *A. calamus* and *L. javanica* completed in ESI+ mode produced (BPI) chromatograms, a wide profile of metabolites eluting over a specified retention time frame, as shown in Figure 3.

The chromatogram of *A. calamus* with the dominating of a particularly intense peak at ∼400 s, with a highest intensity of ∼1.2 × 10^10^. Subsequent moderate peaks were detected at ∼700, ∼550, ∼200, and ∼50 s. Conversely, *L. javanica* offered an evenly dispersed chromatographic profile with multiple peaks of medium to high intensity detected between 100s and 600s. The most intense peak occurred at ∼220 s (∼4.0 × 10^9^). Additional prominent peaks were detected at ∼720, ∼600, ∼400, and ∼100 s.

### Multivariate analysis of *A. calamus* and *L. javanica* data

**FIGURE 4:**
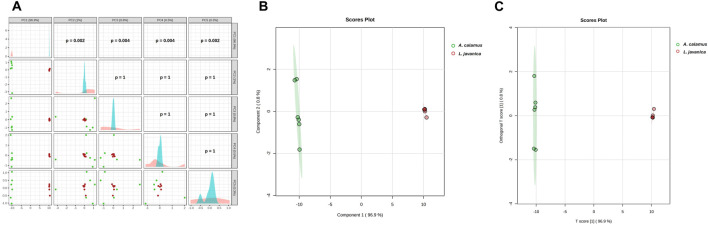
Multivariate analysis: **(A)** Principal Component Analysis (PCA) pairwise scatterplots, **(B)** Partial Least Squares Discriminant Analysis (PLS-DA) scores plot, and **(C)** Orthogonal Partial Least Squares Discriminant Analysis (OPLS-DA) scores plot of *A*. *calamus* (green) and *L. javanica* (red).

Principal Component Analysis (PCA) score model of the associated features displays complete species segregation across PC1, with a variance of 96.9% (PC2 = 1.0%). The PCA pair-plot validates that cluster variances are concentrated on the first component and also noticeable, but smaller on higher components. The dispersals of scores vary significantly for PC2: *p* = 0.002, PC3: *p* = 0.004, PC4: *p* = 0.004, and PC5: *p* = 0.002 (Figure 4A). These results signify the possession of distinct global metabolomic fingerprints of the two species, dominated by variance expressed on PC1. The observed clustering patterns were statistically significantly validated using the PERMANOVA test, consisting of an *F*-value of 936.71, a *p-*value = 0.005, and *R* ^*2*^ = 0.98944.

Moreover, supervised Partial Least Squares-Discriminant Analysis (PLS-DA) generated the same qualitative pattern of PCA score model with distinct separated clusters driven by component 1 (96.9%), though component 2 represented insignificant variance of 0.8% (Figure 4B). Cross-validation metrics also validate with *R* ^*2*^ and *Q* ^*2*^ values that were close to 1.0 for all 5 tested components, which displayed consistently high performance. Moreover, the permutation test additionally validated the PLS-DA model, with a noticeable statistic positioned (*p* < 0.01, 0/100) that was distant outside the permutation distribution (Supplementary Figure S2).

Orthogonal PLS-DA (OPLS-DA) model additionally displays variation into a single predictive component (T score [1] = 96.9%) that segregates the plant species and an orthogonal component (Orthogonal T score [1] = 0.8%) representing within the class structure (Figure 4C). The model was further validated by cross-validation, where the model generated high explanatory and predictive performance, with *R* ^*2*^Y = 1.0, *R* ^*2*^X = 0.969, and *Q* ^*2*^ = 0.999. The related *p*-values for the predictive component (p1) were *R* ^*2*^X: 0.969; *R* ^*2*^Y: 1.0; *Q* ^*2*^: 0.999, whereas for the orthogonal component (o1) were *R* ^*2*^X: 0.00813, *R* ^*2*^Y: 0.00031, and *Q* ^*2*^: 7.04 × 10^-5^, underscoring the model’s explanatory and predictive capability. Further validation of the model was done using a permutation test, with *Q* ^*2*^ and *R* ^*2*^Y values of 0.969 and 0.979, respectively (Supplementary Figure S2).

### Univariate analysis of *A. calamus* and *L. javanica* data

**FIGURE 5:**
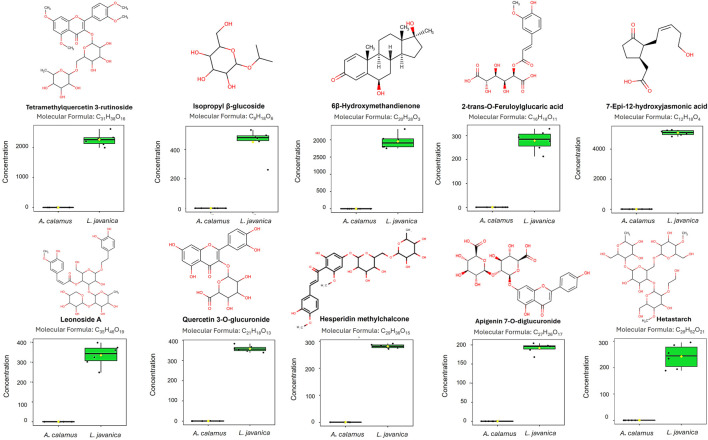
Differential metabolites distinguished between *A. calamus* and *L. javanica*. Molecular formulas, chemical structures, and boxplots of the top ten compounds (VIP >1.5, FC >2.0, *p* < 0.05, FDR-adjusted *p* < 0.05) are shown, with abundances in *A. calamus* and *L. javanica*.

Univariate analysis recognized the top ten metabolites with VIP >2.0, FC >2.0, *p* < 0.05 and FDR-adjusted *p* < 0.05 that significantly differentiated the clusters of *A. calamus* and *L. javanica* (Table 1). Figure 5 depicts major significant metabolites comprising Tetramethylquercetin 3-rutinoside, Isopropyl β-glucoside, and 6β-Hydroxymethandienone, altogether presenting an extreme fold of enrichment. Further significant metabolites comprised of 2-trans-O-Feruloylglucaric acid, 7-Epi-12-hydroxyjasmonic acid, and Leonoside A. Flavonoid derivatives, for instance Quercetin 3-O-glucuronide, Hesperidin methylchalcone, and Apigenin 7-O-diglucuronide were also significantly differentiated. Additionally, Hetastarch also provided a contribution to the metabolic variance. Together, these compounds underscore jasmonates, flavonoid glycosides, and phenolic derivatives as crucial, statistically significant metabolites, supporting the distinct chemical divergence amongst the two plant species. These compounds show higher abundances constantly in *L. javanica* (Figure 5). A detailed chemical profiling of each compound is represented in the Supplementary Table S1.

**TABLE 1:** Differential metabolites distinguished from *A. calamus* and *L. javanica* in electrospray ionization ESI+ mode.

Compound name	RT (min)	Molecular Weight	*m/z*	HMDB_ID	Formula	Log^2^(FC)	T-Test	FDR Log10 (p-adj)	VIP
ALKALOIDS									
5-Isothiocyanatoindane	3,015	115,17	157,1334	CSID642931	C_6_H_13_NO	-6,7198	4,54E-05	4,342703	1,830521
3-Indolehydracrylic acid	3,336	205,0736	206,0809	HMDB0059765	C_11_H_11_NO_3_	5,338881	2,95E-05	4,52994	1,634694
Hydrocotarnine	4,956	221,1048	222,1121	HMDB0033701	C_12_H_15_NO_3_	5,347002	4,08E-08	7,38938	1,6638
Mescaline	2,991	211,26	229,1543	HMDB0254474	C_11_H_17_NO_3_	5,105346	4,88E-05	4,311856	1,602307
Perlolyrine	4,972	264,0895	265,0967	HMDB0030327	C_16_H_12_N_2_O_2_	-5,99531	0,001842	2,73468	1,680894
Prodolic acid	5,075	273,33	296,1276	HMDB0256794	C_16_H_19_NO_3_	-6,27265	0,001789	2,747286	1,732869
CARBOHYDRATES									
Isopropyl β-glucoside	3,5	222,24	245,1016	HMDB0032705	C_9_H_18_O_6_	10,83254	3,52E-05	4,453856	2,331036
Muramic acid	3,206	251,1014	252,1087	HMDB0003254	C_9_H_17_NO_7_	8,105161	9,48E-05	4,023081	2,006866
1-O-Caffeoyl-beta-glucose	5,172	342,0944	343,1016	HMDB0302440	C_15_H_18_O_9_	7,814332	1,86E-06	5,730844	1,977503
Cellulose, microcrystalline	2,156	370,35	393,136	HMDB0032197	C_14_H_26_O_11_	6,787933	8,9E-06	5,050728	1,842234
O-Desmethyltramadol glucuronide	3,259	425,50	448,1957	HMDB0060856	C_21_H_31_NO_8_	-6,19442	2,12E-05	4,673131	1,758105
Persicogenin 3'-glucoside	4,747	456,1638	479,1532	HMDB0041398	C_23_H_26_O_11_	8,080171	1,16E-06	5,934704	2,012623
Hetastarch	4,045	736,70	759,2936	HMDB0253113	C_29_H_52_O_21_	8,836521	2,09E-05	4,679721	2,134599
LIPIDS									
4-Methyl-2-pentenoic acid	4,046	114,14	156,1018	HMDB0031561	C_6_H_10_O_2_	6,668955	6,85E-05	4,164162	1,833963
Undecylenic acid	4,113	184,27	226,1798	HMDB0033724	C_11_H_20_O_2_	7,923542	8,84E-08	7,053706	2,029285
12-Hydroxydodecanoic acid	7,277	216,32	239,1638	HMDB0002059	C_12_H_24_O_3_	-5,60027	1,14E-05	4,941239	1,679658
11-Dodecenoic acid	4,664	198,30	262,1797	HMDB0032248	C_12_H_22_O_2_	6,21621	1,46E-06	5,834954	1,771416
9-Pentadecenoic acid	10,834	240,38	263,2002	HMDB0029765	C_15_H_28_O_2_	-5,45158	0,000253	3,59735	1,674411
Stearidonic acid	9,676	276,40	299,1999	HMDB0006547	C_18_H_28_O_2_	4,57766	1,96E-07	6,707939	1,521083
MG(18:3/0:0/0:0)	8,725	352,2605	353,2678	HMDB0011570	C_21_H_36_O_4_	5,971525	2,8E-07	6,553359	1,761732
Prostaglandin A2	7,707	334,40	357,2028	HMDB0002752	C_20_H_30_O_4_	7,579842	0,000121	3,916245	1,940373
Eicosanedioic acid	10,522	342,50	365,2678	HMDB0242141	C_20_H_38_O_4_	6,161779	5,82E-06	5,23503	1,764398
Thromboxane B3	4,446	368,50	386,2529	HMDB0005099	C_20_H_32_O_6_	-6,81951	0,000823	3,084822	1,814574
MG(5-iso PGF2VI/0:0/0:0)	6,209	400,50	423,2344	HMDB0260485	C_21_H_36_O_7_	6,891431	1,09E-05	4,962081	1,86861
LysoPA(18:2/0:0)	4,868	434,50	457,2398	HMDB0007856	C_21_H_39_O_7_P	6,062893	1,42E-06	5,847397	1,754746
DG(20:4-2OH/0:0/2:0)	8,79	452,60	475,2682	HMDB0297002	C_25_H_40_O_7_	-8,77228	1,31E-05	4,882486	2,094746
DG(2:0/PGD2/0:0)	7,165	468,60	491,2629	HMDB0296907	C_25_H_40_O_8_	-5,07408	0,000151	3,820683	1,583894
DG(6 keto-PGF1alpha/2:0/0:0)	4,095	486,60	528,3155	HMDB0296912	C_25_H_42_O_9_	5,338498	0,000864	3,063593	1,605199
LysoPE(22:2/0:0)	10,093	533,3475	534,3547	HMDB0011522	C_27_H_52_NO_7_P	-6,18444	0,00202	2,694655	1,568301
NITROGEN COMPOUND									
1,3-Hexadien-3-amine	1,032	97,0895	98,09678	CSID67029750	C_6_H_11_N	6,805566	9,38E-06	5,027828	1,856891
2-Aminocyclohexanecarboxylic acid	1,229	143,0945	144,1017	CSID133327	C_7_H_13_NO_2_	5,478209	4,02E-06	5,395528	1,656544
7-Aminomethyl-7-carbaguanine	1,725	179,0804	180,0877	HMDB0011690	C_7_H_9_N_5_O	7,746392	4,59E-06	5,33773	2,019233
5-Fluoromethylornithine	4,624	164,18	187,0863	HMDB0245493	C_6_H_13_FN_2_O_2_	5,535211	8,43E-08	7,073976	1,674376
Succinyl proline	3,047	215,20	238,0682	CSID168469	C_9_H_13_NO_5_	7,81002	9,47E-08	7,02377	1,978274
erythro-4-Hydroxyarginine	3,109	190,20	229,0704	HMDB0034326	C_6_H_14_N_4_O_3_	6,393116	3,43E-07	6,464989	1,81208
Pantothenic acid	9,416	219,23	237,1402	HMDB0000210	C_9_H_17_NO_5_	-6,60822	0,000512	3,290701	1,785286
Phenylalanyl-Glycine	4,839	222,24	245,0917	HMDB0304788	C_11_H_14_N_2_O_3_	4,586087	6,19E-06	5,208442	1,522839
Glutaminylleucine	3,04	259,30	277,1906	HMDB0028801	C_11_H_21_N_3_O_4_	7,790423	1,82E-05	4,74028	1,971685
Glycyl-Tryptophan	3,196	261,279	279,1448	HMDB0028852	C_13_H_15_N_3_O_3_	-6,59597	0,001815	2,741203	1,589188
N-Acetyllactosamine	2,923	383,35	406,1337	HMDB0001542	C_14_H_25_NO_11_	6,16663	0,000286	3,544322	1,745963
NUCLEOSIDE / NUCLEOTIDE									
2-Deoxy-ribono-1,4-lactone	5,407	132,0422	133,0495	HMDB0033958	C_5_H_8_O_4_	4,608802	2,27E-05	4,644565	1,515139
2',3'-Didehydro-2',3'-dideoxycytidine	4,434	209,0811	210,0884	HMDB0245545	C_9_H_11_N_3_O_3_	-8,76604	1,28E-05	4,892618	2,092533
Keto-3-deoxy-manno-octulosonic acid	3,079	238,19	261,0601	HMDB0244292	C_8_H_14_O_8_	6,750766	4,57E-06	5,340459	1,856524
5-(Hydroxymethyl)cytidine	2,821	273,0955	274,1027	CSID32720391	C_10_H_15_N_3_O_6_	4,788076	2,22E-06	5,653688	1,616127
2',3'-Dideoxyadenosine	6,718	235,24	277,1405	HMDB0245544	C_10_H_13_N_5_O_2_	-6,84114	4,18E-05	4,379233	1,84559
5-Methyldeoxycytidine	2,036	241,24	283,1396	HMDB0002224	C_10_H_15_N_3_O_4_	-8,43195	0,00119	2,924626	1,881698
3'-C-Ethynylcytidine	3,174	267,24	309,1174	HMDB0252093	C_11_H_13_N_3_O_5_	8,752045	3,19E-08	7,496043	2,107001
2'-Deoxy-5-formylcytidine	1,99	255,23	319,0993	CSID10291642	C_10_H_13_N_3_O_5_	5,632298	2,15E-06	5,666959	1,744307
Dimp	4,25	332,0524	333,0597	HMDB0006555	C_10_H_13_N_4_O_7_P	8,036495	2,38E-07	6,623169	2,006318
2-Methylguanosine	4,679	297,27	339,1407	HMDB0005862	C_11_H_15_N_5_O_5_	-5,73448	0,000162	3,789346	1,681938
OTHER									
2,4-Pentadienal	10,135	82,04236	83,04964	HMDB0031597	C_5_H_6_O	7,510876	1,41E-06	5,850963	1,943115
5-Methylcytosine	6,097	125,0603	126,0676	HMDB0002894	C_5_H_7_N_3_O	-8,4848	4,81E-06	5,317795	2,059602
xi-4-Hydroxy-4-methyl-2-cyclohexen-1-one	3,795	126,0681	127,0754	HMDB0033629	C_7_H_10_O_2_	8,258472	6,02E-06	5,22045	2,034825
gamma-Butyrolactone	3,78	86,09	128,0706	HMDB0000549	C_4_H_6_O_2_	7,403507	6,82E-06	5,166232	1,925084
Indol-2-one	3,251	131,0371	132,0444	HMDB0253466	C_8_H_5_NO	5,862663	3,16E-07	6,500593	1,718906
Methyl 2-thiofuroate	0,897	142,0115	143,0188	HMDB0037762	C_6_H_6_O_2_S	4,48824	1,48E-05	4,829513	1,511626
Norcamphoric acid	3,195	158,15	200,0915	CSID207959	C_7_H_10_O_4_	5,611922	7,34E-06	5,134455	1,720562
4-Guanidino-1-butanol	0,787	131,18	173,1396	CSID4476579	C_5_H_13_N_3_O	-5,88882	0,001006	2,997208	1,515761
1-Oxo-1H-2-benzopyran-3-carboxaldehyde	4,626	174,0315	175,0388	HMDB0030577	C_10_H_6_O_3_	6,426311	1,56E-05	4,807221	1,825317
3-Hydroxysuberic acid	2,866	190,19	232,1191	HMDB0000325	C_8_H_14_O_5_	4,813224	4,4E-06	5,356159	1,568506
6-(2-Hydroxyethoxy)-6-oxohexanoic acid	3,717	190,19	213,0755	HMDB0061681	C_8_H_14_O_5_	4,69247	4,65E-06	5,332964	1,552665
N-Lactoylphenylalanine	4,391	215,1177	238,1069	HMDB0062175	C_12_H_15_NO_4_	5,606803	5,75E-07	6,240409	1,704328
2-(2-Furylmethyl)-1-indanol	3,905	214,26	237,0907	CSID40514807	C_14_H_14_O_2_	-5,2121	0,000218	3,660803	1,591246
Piperdial	6,936	250,1565	251,1638	HMDB0035798	C_15_H_22_O_3_	-5,12368	4,52E-06	5,345311	1,600008
1-(Ribofuranosyl)indoline	2,641	251,1153	252,1226	CSID67029342	C_13_H_17_NO_4_	4,946661	1,05E-05	4,977423	1,570688
2-Hydroxyacorenone	9,271	236,35	259,1664	HMDB0030916	C_15_H_24_O_2_	-7,68915	1,73E-06	5,761784	1,961428
Risbitin	5,609	222,32	264,1954	HMDB0302980	C_14_H_22_O_2_	-5,92921	0,001049	2,979129	1,703258
Linamarin	1,312	247,24	270,0944	HMDB0033699	C_10_H_17_NO_6_	-6,29487	0,000877	3,056864	1,759135
1-Hydroxyacorenone	7,673	250,33	273,1456	HMDB0030917	C_15_H_22_O_3_	-7,10849	1,82E-06	5,739609	1,887368
4-Hydroxycyclohexylcarboxylic acid	4,619	144,17	289,1637	HMDB0001988	C_7_H_12_O_3_	6,014314	5,86E-06	5,232144	1,73907
Pollenin A	4,254	302,042	303,0493	HMDB0303704	C_15_H_10_O_7_	8,76912	2,16E-07	6,664657	2,126655
9,10-DiHODE	4,767	312,40	330,2632	HMDB0010221	C_18_H_32_O_4_	-4,97006	0,000693	3,159427	1,553509
6β--Hydroxymethandienone	10,138	316,40	339,1922	HMDB0005832	C_20_H_28_O_3_	10,73503	2,66E-06	5,575855	2,317918
Bisoprolol	5,313	325,40	348,2162	HMDB0014750	C_18_H_31_NO_4_	5,110144	2,27E-05	4,643337	1,607036
6'-Hydroxyenterolactone	4,893	314,30	356,1482	HMDB0041697	C_18_H_18_O_5_	6,599311	0,00027	3,568419	1,804397
Caryoptosidic acid	2,971	392,35	415,1204	HMDB0034249	C_16_H_24_O_11_	6,037738	0,000224	3,649573	1,731817
Tyromycic acid	12,521	452,3278	453,3351	HMDB0035888	C_30_H_44_O_3_	-5,71222	4,22E-05	4,374993	1,690313
Fluocinolone	2,966	412,4	454,2062	HMDB0252347	C_21_H_26_F_2_O_6_	-5,03287	0,000493	3,307314	1,561579
Persiconin	4,285	478,1466	479,1539	HMDB0037482	C_23_H_26_O_11_	8,487737	1,03E-06	5,987367	2,064423
Phaseolus epsilon	3,657	526,50	544,2378	HMDB0035039	C_25_H_34_O_12_	7,87868	7,04E-05	4,152464	1,979816
19-Nor-5-androstenediol	9,685	276,40	553,4239	HMDB0004590	C_18_H_28_O_2_	7,24471	8,58E-06	5,066603	1,926738
7-Dehydrologanin tetraacetate	3,488	556,1755	557,1828	CSID391585	C_25_H_32_O_14_	-6,16628	0,003738	2,427367	1,709022
Procyanidin	3,823	578,1412	579,1484	HMDB0013690	C_30_H_26_O_12_	-7,188	0,000208	3,681349	1,883821
Desglucocheirotoxol	13,088	552,70	591,2582	HMDB0033828	C_29_H_44_O_10_	5,752651	2,39E-05	4,621974	1,696989
Neolicuroside	3,869	550,50	592,201	HMDB0040728	C_26_H_30_O_13_	8,370981	5,7E-06	5,243925	2,048135
Marmesin rutinoside	2,724	554,50	596,2325	HMDB0041413	C_26_H_34_O_13_	-6,94662	0,0022	2,657484	1,81168
Hesperidin methylchalcone	4,557	624,2032	625,2106	HMDB0253112	C_29_H_36_O_15_	9,090381	1,04E-08	7,982463	2,139523
Glucoliquiritin apioside	3,01	712,60	730,2684	HMDB0041149	C_32_H_40_O_18_	-6,08309	0,00112	2,950734	1,673043
Leonoside A	4,557	770,70	788,2936	HMDB0040342	C_35_H_46_O_19_	9,611505	1,25E-05	4,904459	2,19735
PHENOLIC									
Coumarin	2,503	146,0365	147,0438	HMDB0001218	C_9_H_6_O_2_	5,452435	0,000132	3,879057	1,64696
3 Hydroxycoumarin	4,771	162,0314	163,0387	HMDB0002149	C_9_H_6_O_3_	4,910609	2,63E-07	6,580547	1,573768
4,8-Dimethyl-7-hydroxycoumarin	3,343	190,19	208,0965	CSID4512230	C_11_H_10_O_3_	5,339346	1,67E-05	4,77794	1,635275
Vanillactic acid	1,727	212,0682	213,0755	HMDB0000913	C_10_H_12_O_5_	7,713922	6,68E-07	6,175003	1,979313
Apigenin	5,177	270,0523	271,0596	HMDB0002124	C_15_H_10_O_5_	5,845909	7,92E-05	4,101263	1,718134
Kaempferol	4,724	286,047	287,0542	HMDB0005801	C_15_H_10_O_6_	7,038508	1,48E-06	5,830917	1,898751
3-Feruloyl-1,5-quinolactone	4,631	350,30	373,091	HMDB0029289	C_17_H_18_O_8_	7,663669	2,15E-05	4,668116	1,956575
2-trans-O-Feruloylglucaric acid	3,606	386,0841	387,0913	HMDB0302546	C_16_H_18_O_11_	9,917521	9,38E-06	5,027846	2,226979
Glucosyloxyanthraquinone	3,844	386,40	409,0911	CSID389045	C_20_H_18_O_8_	-5,23257	0,000103	3,987626	1,611837
Eriodictin	4,559	434,1204	435,1277	HMDB0037480	C_21_H_22_O_10_	8,417998	1,65E-10	9,781596	2,058779
Catechin 7-glucoside	3,177	452,131	453,1383	HMDB0037949	C_21_H_24_O_11_	-5,3561	0,003285	2,483526	1,591291
Quercetin 3-O-glucuronide	4,241	478,0739	479,0812	HMDB0029212	C_21_H_18_O_13_	9,420172	6,67E-08	7,175578	2,186479
Sesaminol glucoside	7,259	532,50	555,1482	HMDB0041209	C_26_H_28_O_12_	4,976722	7,32E-05	4,135256	1,570614
Apigenin 7-O-diglucuronide	4,027	622,1149	623,1222	HMDB0301685	C_27_H_26_O_17_	9,063099	3,86E-07	6,413527	2,131068
Tetramethylquercetin 3-rutinoside	4,052	666,60	708,2469	HMDB0039337	C_31_H_38_O_16_	11,67918	1,57E-06	5,804899	2,418616
POLYKETIDES									
1(2H)-Pentalenone	2,502	118,042	119,0493	CSID66739022	C_8_H_6_O	4,673748	0,000182	3,739442	1,516804
Phthalide	2,503	134,13	157,0282	HMDB0032469	C_8_H_6_O_2_	8,291181	0,000232	3,634062	2,029866
Aflatoxin G2	6,708	330,0731	331,0804	HMDB0030475	C_17_H_14_O_7_	7,294113	0,000298	3,526371	1,900981
TERPENOIDS									
Shinanolone	4,667	192,075	193,0823	HMDB0030580	C_11_H_12_O_3_	-5,65953	0,005135	2,289432	1,589457
4,7-Megastigmadien-9-ol	8,59	194,1668	195,1741	HMDB0038731	C_13_H_22_O	-5,78981	3,46E-05	4,460445	1,700406
Vomifoliol	3,517	224,1409	225,1482	HMDB0303570	C_13_H_20_O_3_	6,685312	3,63E-06	5,439498	1,850491
7-Epi-12-hydroxyjasmonic acid	3,942	226,12	227,1273	HMDB0303749	C_12_H_18_O_4_	9,757035	2,83E-08	7,548945	2,218914
8-Hydroxygeraniol 8-O-glucoside	4,457	332.39	374,2165	HMDB0035025	C_16_H_28_O_7_	5,392225	0,000565	3,248129	1,621572
3beta-Hydroxy-5-cholestenal	9,848	400,60	423,3249	HMDB0060131	C_27_H_44_O_2_	-5,19836	1,33E-05	4,874901	1,62304
7a,12a-Dihydroxy-cholestene-3-one	10,46	416,60	439,3199	HMDB0002197	C_27_H_44_O_3_	-7,53264	7,79E-06	5,108677	1,956674
Limonoate a-ring-lactone	6,644	488,2015	489,2088	HMDB0302537	C_26_H_32_O_9_	-5,00973	0,001182	2,927482	1,561349
Cucumerin A	2,79	552,50	575,1571	HMDB0301967	C_29_H_28_O_11_	6,765172	0,000685	3,164562	1,819729
Cholic acid glucuronide	12,017	584,70	623,2843	HMDB0002577	C_30_H_48_O_11_	4,8136	9,02E-06	5,04476	1,549668
Linalool (8-hydroxydihydro-)	4,627	650,70	692,2884	HMDB0304700	C_32_H_42_O_14_	6,441235	4,01E-05	4,396548	1,794936

Key: 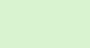 Highlighted top ten differential metabolites distinguished between *A. calamus* and *L. javanica*.

### Pharmacokinetic and drug-likeness activity of differential metabolites

The assessment of the ten most significant metabolites from *A. calamus* and *L. javanica* was conducted through the SWISSADME platform to acquire the ADME profiles of each significant metabolite. This yields essential insights into their potential efficacy as neuroactive inhibitors targeting SH-SY5Y neuroblastoma cells (Table 2).

**TABLE 2:** Physicochemical, pharmacokinetic, drug-likeness, and medicinal chemistry properties of the selected differential bioactive secondary metabolites.

Parameters	Isopropyl β-glucoside	Hetastarch	6β--hydroxymethandienone	Hesperidin methylchalcone	Leonoside A	2-trans-O-Feruloylglucaric acid	Quercetin 3-O-glucuronide	Apigenin 7-O-diglucuronide	Tetramethylquercetin 3-rutinoside	7-Epi-12-hydroxyjasmonic acid
Physicochemical Properties										
Formula	C9H18O6	C29H52O21	C20H28O3	C29H36O15	C35H46O19	C16H18O11	C21H18O13	C27H26O17	C31H38O16	C12H18O4
Molecular weight	222.24 g/mol	736.71 g/mol	316.43 g/mol	624.59 g/mol	770.73 g/mol	386.31 g/mol	478.36 g/mol	622.49 g/mol	666.62 g/mol	226.27 g/mol
No. heavy atoms	15	50	23	44	54	27	34	44	47	16
No. arom. heavy atoms	0	0	0	12	12	10	16	16	12	0
Fraction Csp3	1.00	1.00	0.60	0.48	0.57	0.44	0.24	0.37	0.48	0.50
No. of rotatable bonds	0	15	6	11	13	4	4	7	13	9
No. H-bond acceptors	6	21	3	15	19	11	13	17	16	4
No. H-bond donors	0	11	0	9	11	6	8	9	8	0
Molar Refractivity	49.89	156.69	93.83	148.42	179.64	87.17	110.77	139.71	158.16	61.42
TPSA	55.38 Å^2^	314.83 Å^2^	43.37 Å^2^	245.29 Å^2^	304.21 Å^2^	179.28 Å^2^	227.58 Å^2^	283.34 Å^2^	251.36 Å^2^	52.60 Å^2^
Lipophilicity										
Log Po/w (iLOGP)	3.13	2.21	4.00	2.15	2.28	0.90	1.13	0.28	3.47	3.17
Log Po/w (XLOGP3)	1.84	−7.70	5.49	−0.50	−1.60	−0.99	0.61	−0.24	0.07	2.22
Log Po/w (WLOGP)	2.05	−7.35	4.39	−1.12	−2.27	−1.61	−0.45	−1.94	−0.55	2.01
Log Po/w (MLOGP)	1.50	−7.42	3.48	−2.37	−3.75	−2.23	−2.60	−3.68	−1.99	1.94
Log Po/w (SILICOS-IT)	1.09	−6.09	4.87	−1.14	−2.61	−1.13	−1.04	−2.66	−0.61	2.53
Consensus Log Po/w	1.92	−5.27	4.45	−0.60	−1.59	−1.01	−0.47	−1.65	0.08	2.37
Water solubility										
Log S (ESOL)	−2.38	1.43	−4.86	−2.87	−2.92	−1.62	−3.27	−3.36	−3.35	−2.05
Solubility	9.33e-01 mg/mL; 4.20e-03 mol/L	2.00e+04 mg/mL; 2.71e+01 mol/L	4.32e-03 mg/mL; 1.37e-05 mol/L	8.36e-01 mg/mL; 1.34e-03 mol/L	9.33e-01 mg/mL; 1.21e-03 mol/L	9.24e+00 mg/mL; 2.39e-02 mol/L	2.54e-01 mg/mL; 5.32e-04 mol/L	2.75e-01 mg/mL; 4.41e-04 mol/L	2.99e-01 mg/mL; 4.49e-04 mol/L	2.03e+00 mg/mL; 8.96e-03 mol/L
Class	Soluble	Highly soluble	Moderately soluble	Soluble	Soluble	Very soluble	Soluble	Soluble	Soluble	Soluble
Log S (Ali)	−2.62	1.83	−6.16	−4.18	−4.28	−2.29	−4.96	−5.25	−4.90	−2.96
Solubility	5.29e-01 mg/mL; 2.38e-03 mol/L	4.95e+04 mg/mL; 6.72e+01 mol/L	2.19e-04 mg/mL; 6.94e-07 mol/L	4.09e-02 mg/mL; 6.55e-05 mol/L	4.05e-02 mg/mL; 5.26e-05 mol/L	1.99e+00 mg/mL; 5.14e-03 mol/L	5.20e-03 mg/mL; 1.09e-05 mol/L	3.48e-03 mg/mL; 5.59e-06 mol/L	8.35e-03 mg/mL; 1.25e-05 mol/L	2.48e-01 mg/mL; 1.10e-03 mol/L
Class	Soluble	Highly soluble	Poorly soluble	Moderately soluble	Moderately soluble	Soluble	Moderately soluble	Moderately soluble	Moderately soluble	Soluble
Log S (SILICOS-IT)	−1.45	5.00	−3.83	−0.22	1.04	−0.34	−1.04	0.05	−0.81	−2.55
Solubility	7.89e+00 mg/mL; 3.55e-02 mol/L	7.45e+07 mg/mL; 1.01e+05 mol/L	4.65e-02 mg/mL; 1.47e-04 mol/L	3.77e+02 mg/mL; 6.04e-01 mol/L	8.55e+03 mg/mL; 1.11e+01 mol/L	1.75e+02 mg/mL; 4.52e-01 mol/L	4.36e+01 mg/mL; 9.12e-02 mol/L	6.95e+02 mg/mL; 1.12e+00 mol/L	1.03e+02 mg/mL; 1.55e-01 mol/L	6.35e-01 mg/mL; 2.81e-03 mol/L
Class	Soluble	Soluble	Soluble	Soluble	Soluble	Soluble	Soluble	Soluble	Soluble	Soluble
Pharmacokinetics										
GI absorption	High	Low	High	Low	Low	Low	Low	Low	Low	High
BBB permeant	Yes	No	Yes	No	No	No	No	No	No	Yes
P-gp substrate	No	Yes	No	Yes	No	No	Yes	Yes	Yes	No
CYP1A2 inhibitor	No	No	No	No	No	No	No	No	No	No
CYP2C19 inhibitor	No	No	Yes	No	No	No	No	No	No	No
CYP2C9 inhibitor	Yes	No	Yes	No	No	No	No	No	No	No
CYP2D6 inhibitor	No	No	Yes	No	No	No	No	No	No	No
CYP3A4 inhibitor	No	No	Yes	No	No	No	No	No	No	No
Log Kp (skin permeation)	−6.35 cm/s	−16.26 cm/s	−4.33 cm/s	−10.46 cm/s	−12.14 cm/s	−9.36 cm/s	−8.78 cm/s	−10.27 cm/s	−10.32 cm/s	−6.10 cm/s
Drug likeness										
Lipinski	Yes; 0 violation	No; 3 violations: MW >500, NorO >10, NHorOH >5	Yes; 0 violation	No; 3 violations: MW >500, NorO >10, NHorOH >5	No; 3 violations: MW >500, NorO >10, NHorOH >5	No; 2 violations: NorO >10, NHorOH >5	No; 2 violations: NorO >10, NHorOH >5	No; 3 violations: MW >500, NorO >10, NHorOH >5	No; 3 violations: MW >500, NorO >10, NHorOH >5	Yes; 0 violation
Ghose	Yes	No; 4 violations: MW >480, WLOGP <−0.4, MR >130, #atoms >70	Yes	No; 4 violations: MW >480, WLOGP <−0.4, MR >130, #atoms >70	No; 4 violations: MW >480, WLOGP <−0.4, MR >130, #atoms >70	No; 1 violation: WLOGP <−0.4	No; 1 violation: WLOGP<−0.4	No; 3 violations: MW >480, WLOGP<−0.4, MR >130	No; 4 violations: MW >480, WLOGP <−0.4, MR >130, #atoms >70	Yes
Veber	Yes	No; 2 violations: Rotors >10, TPSA >140	Yes	No; 2 violations: Rotors >10, TPSA >140	No; 2 violations: Rotors >10, TPSA >140	No; 1 violation: TPSA >140	No; 1 violation: TPSA >140	No; 1 violation: TPSA >140	No; 2 violations: Rotors >10, TPSA >140	Yes
Egan	Yes	No; 1 violation: TPSA >131.6	Yes	No; 1 violation: TPSA >131.6	No; 1 violation: TPSA >131.6	No; 1 violation: TPSA >131.6	No; 1 violation: TPSA >131.6	No; 1 violation: TPSA >131.6	No; 1 violation: TPSA >131.6	Yes
Muegge	Yes	No; 5 violations: MW >600, XLOGP3 <−2, TPSA >150, H-acc >10, H-don >5	No; 1 violation: XLOGP3 >5	No; 4 violations: MW >600, TPSA >150, H-acc >10, H-don >5	No; 4 violations: MW >600, TPSA >150, H-acc >10, H-don >5	No; 3 violations: TPSA >150, H-acc >10, H-don >5	No; 3 violations: TPSA >150, H-acc >10, H-don >5	No; 4 violations: MW >600, TPSA >150, H-acc >10, H-don >5	No; 4 violations: MW >600, TPSA >150, H-acc >10, H-don >5	Yes
Bioavailability score	0.55	0.17	0.55	0.17	0.17	0.17	0.11	0.11	0.17	0.55
Medicinal Chemistry										
PAINS	0 alert	0 alert	0 alert	1 alert: catechol_A	1 alert: catechol_A	0 alert	1 alert: catechol_A	0 alert	1 alert: catechol_A	0 alert
Brenk	1 alert: peroxide	0 alert	1 alert: isolated_alkene	2 alerts: catechol, michael_acceptor_1	2 alerts: catechol, michael_acceptor_1	1 alert: cumarine	1 alert: catechol	0 alert	3 alerts: catechol, michael_acceptor_1, more_than_2_esters	2 alerts: mchael_acceptor_1, more_than_2_esters
Leadlikeness	No; 1 violation: MW 250	No; 2 violations: MW >350, Rotors >7	No; 1 violation: XLOGP3 >3.5	No; 2 violations: MW >350, Rotors >7	No; 2 violations: MW >350, Rotors >7	No; 1 violation: MW >350	No; 1 violation: MW >350	No; 1 violation: MW >350	No; 2 violations: MW >350, Rotors >7	No; 2 violations: MW < 250, Rotors >7
Synthetic accessibility	4.34	8.06	4.72	6.37	7.45	5.05	5.26	6.26	6.57	2.31

#### Physicochemical properties

There was a considerable amount of variability in molecular weight, polarity, and solubility of the ten-highest ranked metabolites, which was determined using the SWISSADME. Smaller molecules such as 7-epi-12-hydroxyjasmonic acid (MW 226.27 g/mol) and isopropyl β-glucoside (MW 222.24 g/mol) had a low topological polar surface area (TPSA <60 Å^2^) and the absence of hydrogen bond donors, indicating a pronounced potential for passive permeability across biological membranes, and these molecules also met the physicochemical criteria for drug-like entities. In contrast, more complex glycosylated flavonoids, such as apigenin 7-O-diglucuronide (MW 622.49 g/mol) and Leonoside A (MW 770.73 g/mol), exhibited slightly elevated TPSA (>250 Å^2^) and standout characteristics such as multiple hydrogen bond donors/acceptors, which are conventionally linked to restricted oral bioavailability.

The indices (consensus Log Po/w) for lipophilicity varied from −5.27 for hetastarch to 4.45 for 6β-hydroxymethandienone. This specifically emphasizes the solubility-permeability trade-offs across the metabolite library. Remarkably, 6β-hydroxymethandienone displayed favorable lipophilicity alongside an acceptable molecular weight, implying a significant potential for permeability; conversely, polar glucuronides (e.g., quercetin 3-O-glucuronide, consensus Log Po/w −0.47) are likely impeded in their capacity for cellular penetration.

#### Water solubility

Predictions regarding water solubility indicated that hetastarch is highly soluble (>2.0 × 10^4^ mg/mL), while 6β-hydroxymethandienone displayed suboptimal solubility (4.32 × 10^-3^ mg/mL). Intermediate solubility profiles were noted for flavonoid derivatives, such as hesperidin methylchalcone and tetramethylquercetin 3-rutinoside. These findings suggest that formulation strategies may be necessary for highly lipophilic compounds exhibiting poor intrinsic solubility.

#### Pharmacokinetics

Predictions regarding gastrointestinal (GI) absorption depicted that 6β-hydroxymethandienone, isopropyl β-glucoside, and 7-epi-12-hydroxyjasmonic acid demonstrate high absorption potential, whereas the majority of glycosylated flavonoids exhibited low absorption due to their extensive polar surface areas. Blood-brain barrier (BBB) permeability was anticipated for the smaller hydrophobic compounds, particularly isopropyl β-glucoside, 6β-hydroxymethandienone, and 7-epi-12-hydroxyjasmonic acid, implying a potential for central nervous system (CNS) activity against SH-SY5Y neuroblastoma cells. Significantly, numerous compounds, including hesperidin methylchalcone and apigenin diglucuronide, were identified as substrates for P-glycoprotein (P-gp), suggesting a vulnerability to efflux and diminished intracellular accumulation.

Skin permeation (Log Kp) values were more favorable for lipophilic scaffolds (e.g., −4.33 cm/s for 6β-hydroxymethandienone) in comparison to highly polar compounds (−16.26 cm/s for hetastarch). The analysis of Cytochrome P450 (CYP) inhibition identified 6β-hydroxymethandienone as a multi-isoform inhibitor (CYP2C19, CYP2C9, CYP2D6, CYP3A4), indicating potential drug-drug interaction liabilities, whereas the majority of glycosides exhibited no alerts for CYP inhibition.

#### Drug-likeness

Filters for Drug-likeness indicated that only isopropyl β-glucoside, 6β-hydroxymethandienone, and 7-epi-12-hydroxyjasmonic acid fully conformed to Lipinski’s rule of five, as well as Veber, Egan, and Muegge criteria, while the majority of glycosides surpassed thresholds for hydrogen bonding, molecular weight, and TPSA. Bioavailability scores corroborated these findings, revealing favorable values of 0.55 for the smaller drug-like molecules in contrast to ≤0.17 for the larger glycosides. These outcomes underscore isopropyl β-glucoside and 6β-hydroxymethandienone as promising candidates for oral bioavailability.

#### Medicinal chemistry filters

The identification of Brenk alerts, as well as PAINS (Pan-Assay Interference Compounds), has highlighted various flavonoid derivatives (such as hesperidin methylchalcone, Leonoside A, and quercetin 3-O-glucuronide) that exhibit Michael acceptor motifs or catechol, thereby indicating the likelihood of assay interferences. The synthetic accessibility scores exhibited a range from 2.31 (7-epi-12-hydroxyjasmonic acid, which is readily synthesizable) to 8.06 (hetastarch, which is characterized by high complexity), reflecting considerations of feasibility for prospective lead optimization endeavors. Lead-likeness was attained solely for 6β-hydroxymethandienone, while the remaining metabolites were hindered by excessive molecular dimensions or flexibility.

Therefore, the ADME profiling has prioritized three metabolites, namely isopropyl β-glucoside, 6β-hydroxymethandienone, and 7-epi-12-hydroxyjasmonic acid, which emerge as the most promising lead-like candidates for targeting of SH-SY5Y neuroblastoma cells. Larger polar flavonoid glycosides, despite their bioactivity, may necessitate structural simplification or the implementation of advanced delivery systems to mitigate challenges related to poor absorption and permeability. Their favourable oral bioavailability, blood-brain barrier permeability, and adherence to drug-likeness criteria substantiate their potential for subsequent preclinical validation.

## Discussion

This study validates that extracts of *L. javanica* and *A. calamus* alter mitochondrial functional status and exert dose-dependent cytotoxic effects in SH-SY5Y neuroblastoma cells. The observed shifting toward increased mitochondrial membrane polarization, as evaluated by JC-1 staining, implies preservation or enrichment of mitochondrial reliability at levels of the tested exposure, rather than clear mitochondrial depolarization. Such effects are consistent with complex phytochemical concoctions in which mitochondrial responses might vary depending on exposure duration, concentration, and cellular context [14, 52].

Untargeted metabolomics analysis facilitated the chemical delineation of the phenotypic expressions of substances. With multivariate statistics verifying the distinct chemical differences between the two species, the metabolomic profiling identified a wide range of bioactive substances, including alkaloids, phenolics, flavonoid glycosides, and jasmonates. Separated and distinct groupings were exhibited by *L. javanica* and *A. calamus* in PCA, PLS-DA and OPLS-DA, with cross-validation by OPLS-DA and permutation analyses corroborating the non-random nature of the segregation of classes [53]. Given the propensity for supervised models to exhibit overfitting traits, the interpretation of data of the nearly perfect *R* ^*2*^/*Q* ^*2*^ values with circumspection, anchoring conclusions within the framework of unsupervised PCA delineation and independent univariate filters (VIP, fold-change, *p* values), which is indicative of optimal methodological procedures [54]. The principal discriminative compounds identified were isopropyl β-glucoside, 6β-hydroxymethandienone, 7-epi-12-hydroxyjasmonic acid, 2-trans-O-feruloylglucaric acid, and flavonoid glycosides (e.g., conjugates of quercetin and apigenin) [55]. The identification of a jasmonate (7-epi-12-hydroxyjasmonic acid) is of significant mechanistic relevance; most jasmonates and their derivatives possess the capability to induce cancer cell apoptosis through the disassociation of hexokinase II from the mitochondria, inadvertently, disrupting metabolic processes [56, 57], and multiple research investigations have documented ROS-mediated apoptotic responses across several tumor models [57, 58]. If jasmonate-like chemistry was enriched in *L. javanica*, the plant may contribute to cytotoxic effects at elevated concentrations, notwithstanding the net mixture’s antioxidative behavior under the experimental conditions employed [35, 36].

The chemistry of *L. javanica* varies by chemotype (piperitenone-, carvone-, linalool, or myrcenone-rich), which can modulate biological effects [59]. The metabolomic analysis confirms the divergence of composition from *A. calamus* and focuses on phenylpropanoid, flavonoid, or jasmonate classes for fractionation [33]. The profile for *A. calamus* inherently invokes concerns pertaining to translation, although β-asarone and some crude extracts have the potential to exhibit antiproliferative activity both *in vitro* and *in vivo* [60–62]. β-asarone has been identified as a carcinogenic and genotoxic component in rodents as well as hepatotoxic in preclinical models; this necessitates the establishment of regulatory limits in phytomedicine as well as food products [62]. Consequently, any trajectory for the development of products must prioritize asarone-depleted fractions or alternatives devoid of asarone if the exploration of efficacy is to be conducted safely [42, 43, 62]. It is crucial to note that putative annotations of mescaline-and steroid-like compounds probable indicate structurally associated plant-derived analogues sharing common triterpenoid or phenethylamine biosynthetic scaffolds, instead of definitive identification of canonical reference molecules [48, 63].

Accordingly, SwissADME analysis was employed to provide functional prioritization of annotated metabolites. The SwissADME software was utilized alongside complementary cheminformatics principles (Lipinski, Veber, Ghose, Egan, Muegge), and in conjunction with gastrointestinal absorption/BBB predictions and alert panels (PAINS, Brenk, lead-likeness), this was done to examine the most discriminative metabolites [64–66]. Compounds such as isopropyl β-glucoside, 6β-hydroxymethandienone, and 7-epi-12-hydroxyjasmonic were identified through the internal ADME heatmap as advantageous lead-like candidates that are grounded in minimal rule infractions, bioavailability assessments, and feasible synthetic accessibility [44]. Although SwissADME’s BOILED-Egg framework serves as an ideal tool for preliminary evaluation for BBB and gastrointestinal predictions, further orthogonal verification is usually required through experimental assays such as PAMPA, MDCK/P-gp transport, and microsomal stability as described in this study [47, 67]. Within this limitation-aware framework, *in silico* ADME predictions still serve as a critical early-stage filter for selecting candidate phytometabolites with translational possibility in neuroblastoma research [68]. Predicted BBB permeability is mostly applicable for neuroblastoma, given the connection of paraspinal, intracranial, or central nervous system-adjacent disease locations, where incomplete tissue penetration can restrict therapeutic efficacy [44]. Moreover, predicted P-glycoprotein (P-gp) substrate status alerts the probability of active efflux, which might decrease intracellular accumulation and influence multidrug resistance [69]. Lastly, cytochrome P450 (CYP) inhibition profiles present understanding into possible metabolic liabilities and drug-drug interaction risks, which are important considerations in pediatric oncology settings where integration therapies are common [70]. In general, these parameters reinforce rational prioritization of phytometabolites for downstream optimization, while emphasizing compounds that might need structural refinement or different delivery approaches.

Natural products remain significantly important as complementary or primary scaffolds for neuroblastoma-based therapies, particularly polyphenols, which are a class of secondary metabolites known to influence apoptosis activity and autophagy, and may also specifically target neuroblastoma stem-like cell populations [71, 72]. A compilation of recent scholarly reviews has systematically outlined a variety of phytochemicals exhibiting preclinical activity against neuroblastoma and highlighted proposed combinations of probable strategies aimed at augmenting responses to standard therapeutic regimens [73]. Within this body of literature, our findings motivate a chemotype-specific follow-up: (i) to deprioritize asarone-rich fractions of *A*. *calamus* or to specifically eliminate β-asarone before experimental testing due to safety concerns [62], (ii) to assess and evaluate flavonoid conjugates for their potential to modulate redox status and kinase signaling pathways in neuroblastoma [74], and (iii) to extract jasmonate-like metabolites from *L. javanica* for the purpose of investigating the direct targeting of mitochondrial pathways [57].

Together, these results offer a rationale for structured future investigations that incorporate chemotype-informed isolation approaches, bioactivity-guided fractionation, and target-focused mechanistic assessment.

### Future directions

Building on potential chemotypic signatures identified by incorporating univariate and multivariate metabolomic analyses, future work should prioritize targeted isolation of potential metabolites from dominant chemical classes. Bioactivity-guided fractionation methods can be critical to connect specific phytochemical fractions to functional effects studies in neuroblastoma cells and to isolate active constituents from complex extract mixtures. In silico structure-based molecular docking and molecular dynamics can be used to screen relevant molecular targets against neuroblastoma. To support rational prioritization of candidate chemotypes and guide hypothesis-driven mechanistic investigations.

## Conclusion

This study employed an integrated workflow of untargeted metabolomics, mitochondrial functional assessment, and *in silico* ADME to explore phytometabolites from *L. javanica* and *A. calamus* in human SH-SY5Y neuroblastoma cells. Univariate and multivariate investigations uncovered distinct chemotypic signatures, with *L. javanica* characterized by flavonoid- and polyphenol-enriched profiles and *A. calamus* was dominated by jasmonate-, phenylpropanoid- and lipophilic terpenoid-associated metabolites. Extract exposure related to dose-dependent reductions in cell viability and modulation of mitochondrial membrane potential. In silico pharmacokinetic predictions supported early-stage prioritization of selected chemotypes such as isopropyl β-glucoside, 6β-hydroxymethandienone, and 7-epi-12-hydroxyjasmonic acid. These results are preliminary and hypothesis-generating, and further mechanistic confirmation and *in vivo* studies are recommended to establish biological mechanisms and translational importance.

## Data Availability

The original contributions presented in the study are included in the article/Supplementary Material, further inquiries can be directed to the corresponding author.

## Footnotes

1
www.hmdb.ca

2
www.chemspider.com

3
www.massbank.jp

4
http://www.swissadme.ch/index.php

5
https://www.metaboanalyst.ca
