# Supplementary material for: Metabolomics-guided identification of bioactive phytometabolites from South African plants targeting neuroblastoma
Source: Exp Biol Med (Maywood). 2026 Mar 5;251:10867. doi: 10.3389/ebm.2026.10867 (PMC13001226; doi:10.3389/ebm.2026.10867)
Supplement: Supplementary file 1 [file Supplementaryfile1.docx]

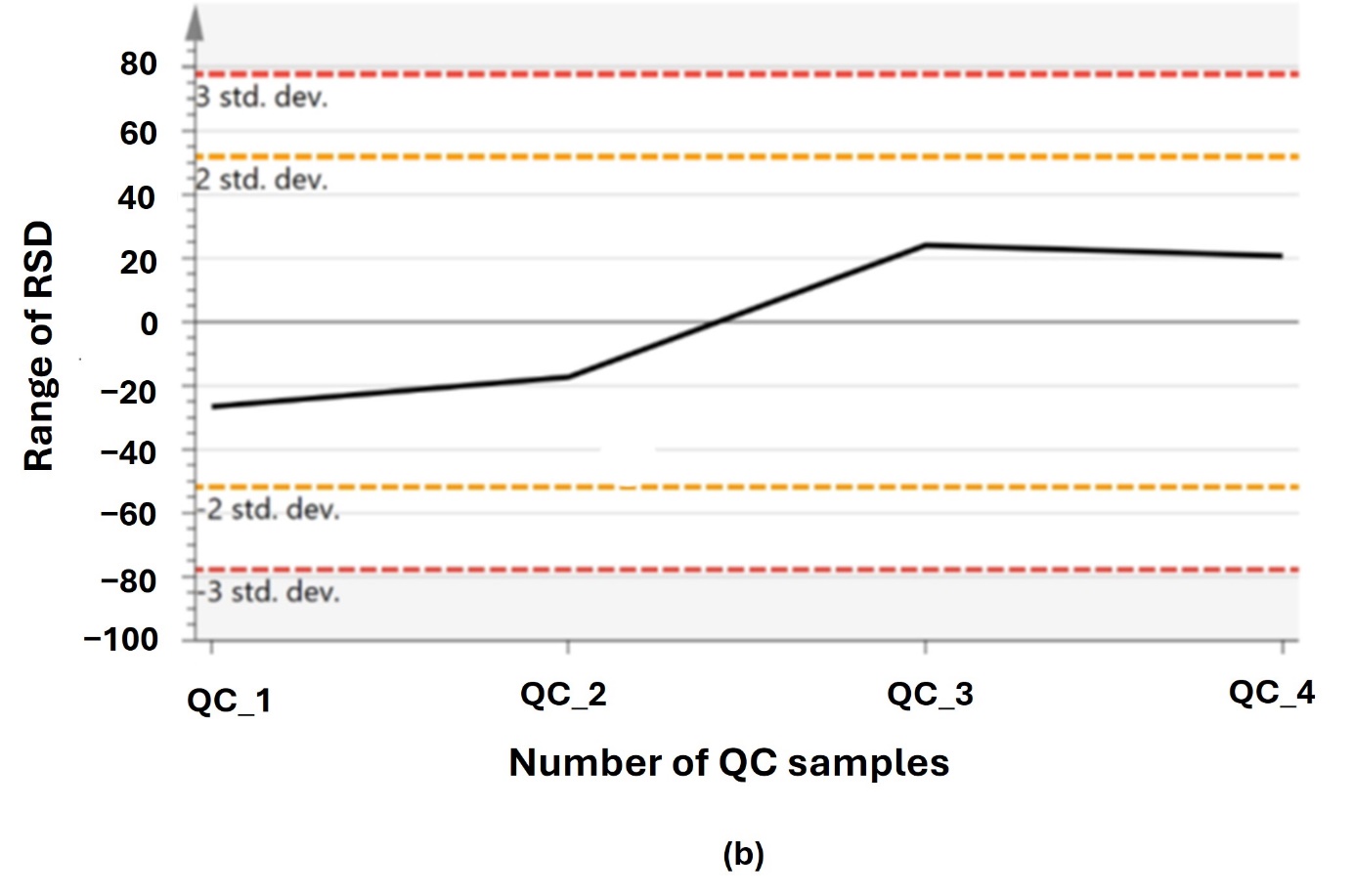


Figure 1S. Quality control samples (QC) plot displaying relative standard deviation (RSD) difference across QC samples (QC_1-QC_4).
