# Supplementary material for: Metabolomics-guided identification of bioactive phytometabolites from South African plants targeting neuroblastoma
Source: Exp Biol Med (Maywood). 2026 Mar 5;251:10867. doi: 10.3389/ebm.2026.10867 (PMC13001226; doi:10.3389/ebm.2026.10867)
Supplement: Supplementary file 3 [file Supplementaryfile2.docx]

**(A)**


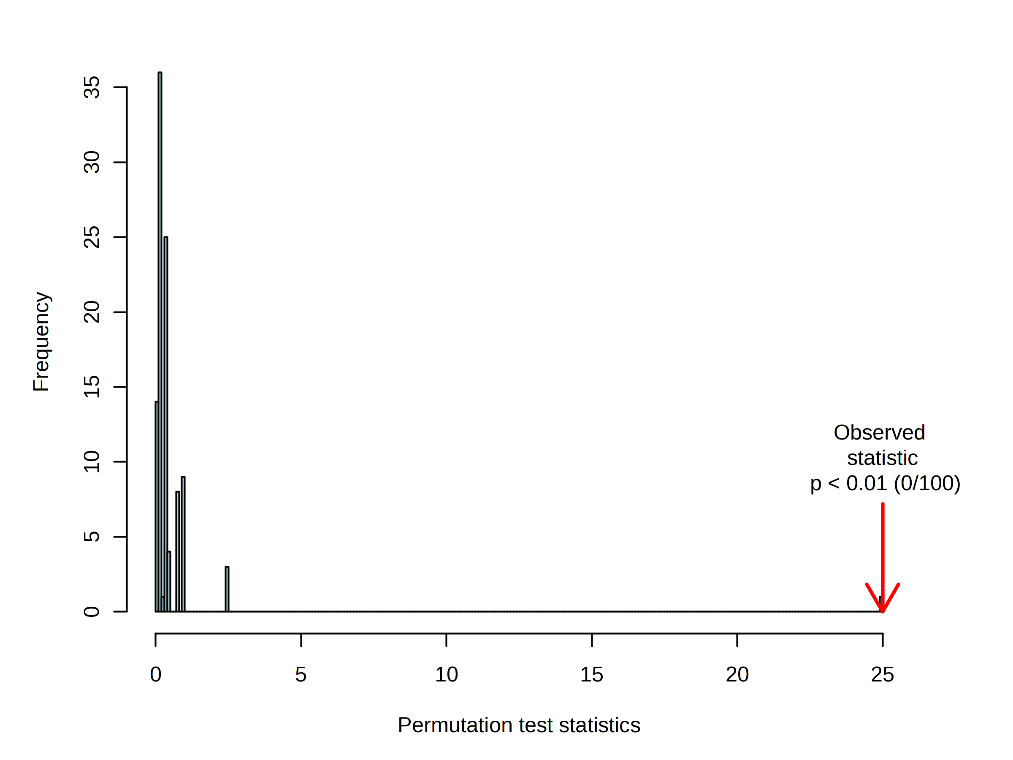


**(B)**


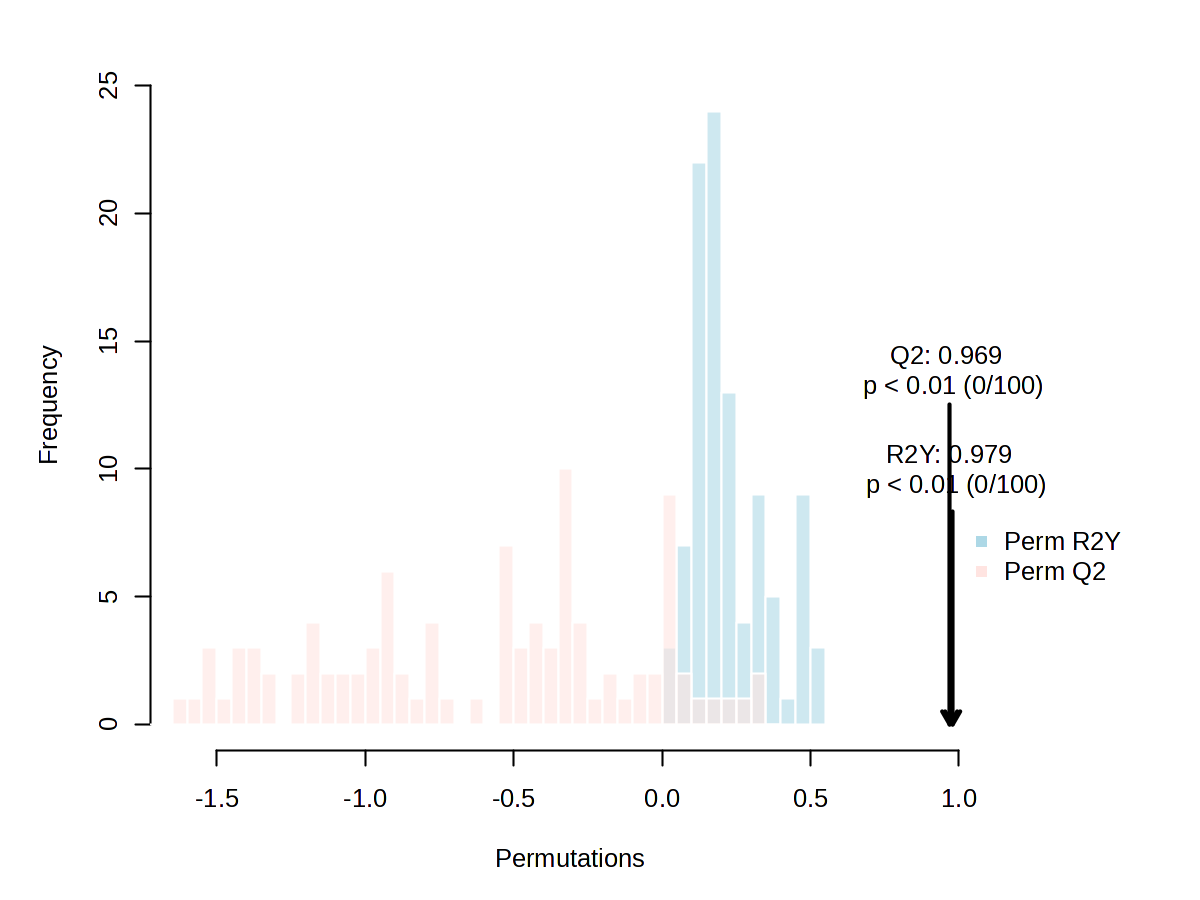


Figure 2S. Permutation testing of the **(A)** PLS-DA and **(B)** OPLS-DA models (n= 100 permutations). The original models (rightmost points) display higher R² and Q² values than all permuted models, with negative Q² intercepts, verifying the lack of model overfitting.
